# Forecasting a Short-Term Photovoltaic Power Model Based on Improved Snake Optimization, Convolutional Neural Network, and Bidirectional Long Short-Term Memory Network

**DOI:** 10.3390/s24123897

**Published:** 2024-06-16

**Authors:** Yonggang Wang, Yilin Yao, Qiuying Zou, Kaixing Zhao, Yue Hao

**Affiliations:** College of Information and Electrical Engineering, Shenyang Agricultural University, Shenyang 110866, China; wygvern@syau.edu.cn (Y.W.); yaoyilin@stu.syau.edu.cn (Y.Y.); kaixingzhao@stu.syau.edu.cn (K.Z.); haoyue@stu.syau.edu.cn (Y.H.)

**Keywords:** PV power generation, convolutional neural network, K-means clustering, improve snake optimization algorithm, bidirectional long short-term memory network

## Abstract

The precision of short-term photovoltaic power forecasts is of utmost importance for the planning and operation of the electrical grid system. To enhance the precision of short-term output power prediction in photovoltaic systems, this paper proposes a method integrating K-means clustering: an improved snake optimization algorithm with a convolutional neural network–bidirectional long short-term memory network to predict short-term photovoltaic power. Firstly, K-means clustering is utilized to categorize weather scenarios into three categories: sunny, cloudy, and rainy. The Pearson correlation coefficient method is then utilized to determine the inputs of the model. Secondly, the snake optimization algorithm is improved by introducing Tent chaotic mapping, lens imaging backward learning, and an optimal individual adaptive perturbation strategy to enhance its optimization ability. Then, the multi-strategy improved snake optimization algorithm is employed to optimize the parameters of the convolutional neural network–bidirectional long short-term memory network model, thereby augmenting the predictive precision of the model. Finally, the model established in this paper is utilized to forecast photovoltaic power in diverse weather scenarios. The simulation findings indicate that the regression coefficients of this method can reach 0.99216, 0.95772, and 0.93163 on sunny, cloudy, and rainy days, which has better prediction precision and adaptability under various weather conditions.

## 1. Introduction

The excessive extraction and consumption of fossil fuels have led to dire environmental pollution. Renewable energy sources, encompassing solar power, biomass energy, wind energy, and hydropower, have witnessed extensive development and utilization. Among numerous renewable energy sources, photovoltaic (PV) power generation holds great importance in ensuring the security, stability, and cost-effective functioning of the electricity system. However, PV power generation exhibits strong randomness and fluctuations that have the potential to significantly disrupt the power grid during large-scale grid integration, ultimately affecting the stability and safety of the power system [1]. Accurate PV power forecasting can mitigate its impact on the electrical grid. Therefore, enhancing the precision of PV forecasting is vital for bolstering the reliability of solar power generation and developing grid scheduling plans.

On the basis of distinct time scales, PV power output forecasting is primarily categorized as long-term, medium-term, and short-term predictions [2]. The long-term forecast can be utilized to evaluate the quarterly and annual power generation indicators of power plants and the tasks of power generation, transmission, and power system distribution [3]; medium-term forecasts are mainly used for the maintenance of electrical systems and PV power plants [4]; and short-term prediction is beneficial for power sector staff to make generation plans quickly and arrange grid dispatching reasonably [5]. Due to the significant importance of short-term solar PV power prediction in providing daily power generation planning decisions for the power industry, and achieving efficient and economic dispatch, it has emerged as a focal point of current research.

Currently, the primary research methodologies for PV power forecasting can be classified into physical methods, statistical methods, and hybrid methods [6,7,8]. The physical method calculates the process and principle of PV power generation through physical formulas such as the solar radiation transfer equation. It involves building a physical model and utilizing environmental information, component parameters, and solar irradiance of PV power stations to predict PV power. However, the modeling process using a physics-based approach is complex and cost-intensive, making it unsuitable for short-term forecasting [9]. Compared to physics methods, statistical approaches employ simpler modeling without requiring complex experimental measurements, thereby possessing better accuracy. The statistical method can be classified into two types: traditional statistical models and artificial intelligence approaches. Traditional statistical models comprise time series analysis [10], grey theory [11], regression analysis [12], etc. Prema and Rao used the time series algorithm to forecast solar power generation, tested the data with different durations, and finally compared the error of the experimental results [13]. Zhong et al. proposed a multidimensional grey prediction algorithm, exhibiting better predictive accuracy compared to conventional grey models [14]. Reikard successfully employed an autoregressive model to predict PV power generation, achieving remarkable performance [15]. The aforementioned approach has demonstrated satisfactory performance in predicting stationary time series. However, solar irradiance is influenced by clouds and seasons, resulting in non-stationary behavior in time series data. Therefore, these models fail to accurately capture the nonlinearity present in the data, leading to subpar predictive capabilities [16].

To address the aforementioned problems, many researchers have commenced employing artificial intelligence approaches [17], for instance, support vector machines [18], extreme learning machines [19], and neural networks [20] for PV power forecasting. Li et al. employed the SVM model for short-term PV power forecasting [21]. Nevertheless, the SVM relies on quadratic programming to determine the support vectors, leading to prolonged training time when dealing with a large number of samples. Al-Dahidi et al. utilized the ELM model to predict PV power [22]. Although this method achieves satisfactory prediction results, the random initialization of weights and biases for hidden layer nodes in the ELM algorithm led to instability and overfitting issues [23]. Kim et al. utilized LSTM to predict ultra-short-term PV power [24]. This approach demonstrates excellent prediction accuracy when applied to large-scale temporal data sequences. However, determining the parameters of the LSTM model can be problematic, as it may not achieve the desired results when applied to other real-world prediction problems.

The hybrid method can leverage the advantages of different single prediction models, ultimately resulting in better predictive efficacy when compared to utilizing a single forecasting method [25,26]. Liu et al. used LSTM to predict PV power and built a LSTM prediction model combined with the dragonfly algorithm (DA) [27]. The experimental outcomes demonstrate that the DA–LSTM model exhibits better predictive accuracy compared to both conventional predictive models and the LSTM model. Zheng et al. established a model for PV power prediction [28]. This innovative approach harnessed particle swarm optimization (PSO) to effectively optimize LSTM networks. The experimental results indicate a noteworthy enhancement in the forecasting precision of the LSTM model after it was optimized with the PSO algorithm. Tuerxun et al. posited an improved condor search (MBES) algorithm to address the issue of selecting the best hyperparameters for LSTM and established an innovative MBES–LSTM model for predicting short-term power [29]. The empirical findings indicate that the MBES–LSTM model surpasses the LSTM model in prediction precision. These documents primarily combine LSTM models with swarm intelligence optimization algorithms to form hybrid models to enhance the precision of power prediction.

Recently, an escalating multitude of scholars have amalgamated multiple deep learning models into a hybridized model with the intent of augmenting the precision of model predictions. For instance, Lim et al. established a hybrid approach composed of a convolutional neural network (CNN) and LSTM [30]. The simulation findings demonstrate that the CNN–LSTM model exhibits favorable predictive performance. When the input temporal sequence expands in length, the information in the sequence is prone to loss, resulting in low prediction precision of the model. He et al. contemplated the bidirectional flow of information and employed a bidirectional long short-term memory network (BiLSTM) for prediction [31]. By integrating the advantages of both the CNN and BiLSTM, a CNN–BiLSTM solar power prediction model is constructed. The CNN was utilized to extract influential factors’ features, while BiLSTM was employed for chronological prediction. The outcomes demonstrate that this approach effectively reduced training time and outperformed traditional forecasting models.

Through a review of the existing literature, it can be found that the current mainstream method is to combine different models to build a hybrid prediction model, but there is a scarcity of literature focusing on leveraging intelligent optimization algorithms to ascertain the optimal parameters of the hybrid model. Taking the CNN–BiLSTM model as an example, this model improves prediction precision, but it has excessive internal parameters and improper selection may lead to potential overfitting issues. The setting of the learning rate, regularization coefficient, and number of hidden layer neurons directly affects the accuracy of PV power prediction results. The learning rate exerts a significant influence on the training effectiveness of the model, while the regularization coefficient is employed to regulate the complexity of the model, thus preventing overfitting. The number of hidden layer neurons plays a pivotal role in the model’s fitting degree, and these parameters have great randomness. Relying solely on human professional knowledge and historical experience to select parameters cannot guarantee the predictive efficacy of the model. Therefore, it is necessary to choose an appropriate optimization algorithm to combine with the CNN–BiLSTM model to acquire the optimal parameters of the CNN–BiLSTM model. Hence, the snake optimization algorithm is introduced to optimize the parameters of the CNN–BiLSTM prediction model, thereby building a novel short-term PV forecasting model.

The snake optimization (SO) algorithm, motivated by principles of biomimetics, was proposed by Hashim and Hussien in 2022 [32]. The SO algorithm possesses advantages such as fast convergence, strong exploitation capability, and minimal parameter adjustments, making it suitable for optimizing the CNN–BiLSTM model. However, the SO algorithm also suffers from the drawback of getting trapped in local optima, which affects its optimization effectiveness. Therefore, this study proposes a multi-strategy improved snake optimization (MISO) algorithm, aiming to avoid the algorithm getting trapped in local optima, bolstering its exploratory capacity, enhancing solution accuracy, and effectively tackling the drawbacks of the original algorithm. In addition, the MISO algorithm proposed in this article is applied to optimize the parameters of the CNN–BiLSTM model and the application of the MISO–CNN–BiLSTM model for predicting PV power. The main contributions of this study are as follows:

(1) K-means clustering is employed to categorize weather patterns into sunny, cloudy, and rainy for the reduction of the impact of data fluctuations on forecasts. Then, a Pearson correlation analysis is conducted on the historical PV data and meteorological factors that exhibit a high correlation with the power sequence are selected as input data for the predictive model.
(2) This study proposes a multi-strategy improved snake optimization (MISO) algorithm, which incorporates multiple optimization strategies to overcome the limitations of the original algorithm. The primary innovations of this approach encompass the subsequent elements: firstly, introducing Tent chaotic mapping to augment the initial population quality of the algorithm; secondly, improving the food quantity threshold to enhance the algorithm’s convergence speed; then, introducing the lens imaging backward learning strategy to enable the algorithm to obtain dynamic and inverse solutions in lens backward learning, further augmenting the algorithm’s optimization prowess; and finally, introducing the optimal individual adaptive disturbance strategy to reduce the possibility of the algorithm getting trapped in local optima.
(3) The optimization performance of the MISO algorithm is evaluated utilizing six classic test functions and compared with the grey wolf optimizer (GWO), whale optimization algorithm (WOA), and SO algorithms. The simulation findings indicate that the MISO algorithm outperforms other basic algorithms in convergence and solution precision. Next, the MISO algorithm and CNN–BiLSTM model are combined to establish the MISO–CNN–BiLSTM PV prediction model. Validated with real historical data from a specific location in Ningxia, China, the proposed method exhibits good precision under sunny, cloudy, and rainy scenarios.

The remaining sections of this paper are as follows: Section 2 introduces the PV power prediction model and multi-strategy improved snake optimization algorithm. Section 3 elucidates the principles of the K-means clustering algorithm, analyzes the factors influencing PV power generation, and identifies model inputs. Section 4 provides an analysis and discussion of the findings from the simulation experiment. Finally, Section 5 provides the conclusion of this study.

## 2. Prediction Model of Photovoltaic Power

### 2.1. Convolutional Neural Network–Bidirectional Long Short-Term Memory Network

#### 2.1.1. Convolutional Neural Network

**Figure 1:**
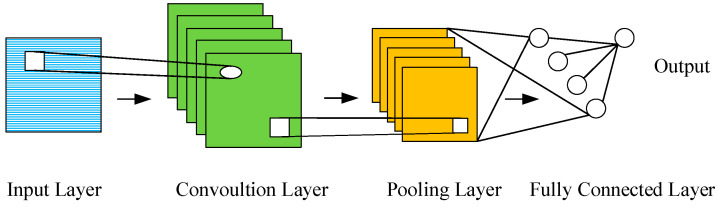
The structure of CNN.

A CNN is primarily utilized for image processing but can also be employed for time series analysis [33]. The CNN mainly consists of convolutional layers and pooling layers, as depicted in Figure 1.

The convolutional layer plays a pivotal role in the architecture of a CNN. It convolves input data with multiple different convolution kernels and extracts features through convolution operation. The convolution process can be expressed as Equation (1).

(1) $$C_{i}=f(x⊗w_{i}+b_{i})$$

where x represents the input of the CNN; $C_{i}$ refers to the *i*-th local feature of the convolutional layer output; ⊗ symbolizes the convolutional operation; and $w_{i}$ and $b_{i}$ are the weight matrix of the *i*-th layer and the bias matrix, respectively.

In order to prevent overfitting, this study adopts the *Relu* activation function, as depicted in Equation (2).

(2) $$f\left(z\right)=ReLu\left(z\right)=\left\{\begin{array}{c} 0,\;\;\;if\;z\leq 0\\ z,\;\;if\;z>0 \end{array}\right.$$

#### 2.1.2. Long Short-Term Memory Network

**Figure 2:**
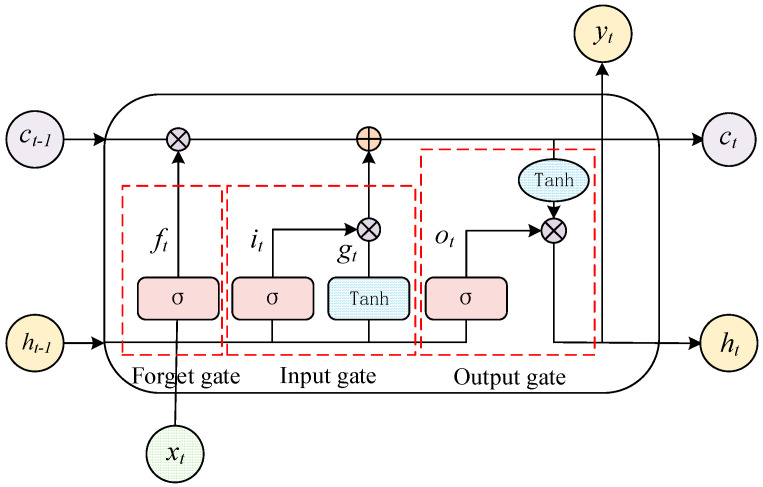
The construction of the LSTM network.

An LSTM network effectively mitigates the issues of gradient vanishing and explosion that plague traditional RNNs during the training of lengthy sequences. As illustrated in Figure 2, the storage unit of an LSTM network is composed of forget gates, input gates, and output gates. The precise computational procedures of LSTM can be elucidated by the subsequent equation [34].

(3) $$f_{t}=\sigma \left[w_{f}\cdot \left(h_{t-1},x_{t}\right)+b_{f}\right]$$

(4) $$i_{t}=\sigma \left[w_{i}\cdot \left(h_{t-1},x_{t}\right)+b_{i}\right]$$

(5) $$g_{t}=tanh\left[w_{g}\cdot \left(h_{t-1},x_{t}\right)+b_{g}\right]$$

(6) $$c_{t}=f_{t}\cdot c_{t-1}+i_{t}\cdot g_{t}$$

(7) $$o_{t}=\sigma \left[w_{o}\cdot \left(h_{t-1},x_{t}\right)+b_{0}\right]$$

(8) $$h_{t}=o_{t}\cdot tanh(c_{t})$$

where σ indicates the activation function; w and b indicate the weight matrix and bias vector of the control gate, respectively; and $h_{t}$ represents the final output result.

#### 2.1.3. Bidirectional Long Short-Term Memory Network

**Figure 3:**
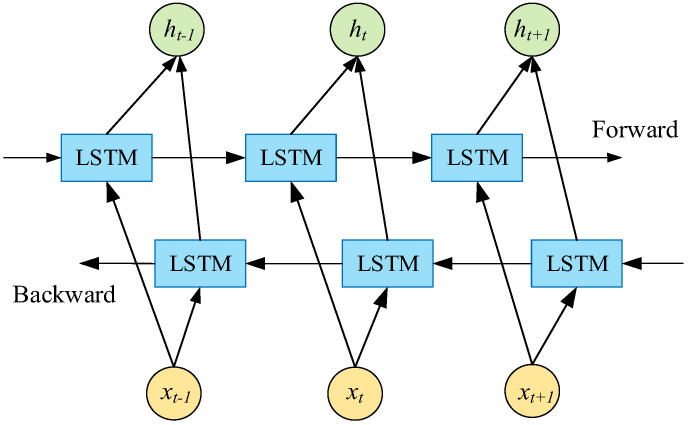
The schematic diagram of BiLSTM neural network.

LSTM neural networks can only train input sequences in one direction and can only consider historical information, resulting in relatively limited data features. However, BiLSTM neural networks can analyze PV data in both directions, comprehensively considering both historical and future information of the data [35]. This improves the comprehensiveness of the forecasting process and enhances the precision of PV power forecasting. The BiLSTM schematic diagram is delineated in Figure 3, while the computation equation is presented below:

(9) $$\vec{h_{t}}=LSTM(h_{t-1},x_{t}{,c}_{t-1})$$

(10) $$\overset{\leftarrow }{h_{t}}=LSTM(h_{t+1},x_{t}{,c}_{t+1})$$

(11) $$h_{t}=\alpha \vec{h_{t}}+\beta \overset{\leftarrow }{h_{t}}$$

where $x_{t}$ represents the input data at time t, $\vec{h_{t}}$ and $\overset{\leftarrow }{h_{t}}$ represent the output of the forward LSTM and backward LSTM hidden layers, respectively, and α and β are constants which denote the weight values for $\vec{h_{t}}$ and $\overset{\leftarrow }{h_{t}}$.

#### 2.1.4. Convolutional Neural Network–Bidirectional Long Short-Term Memory Network

**Figure 4:**
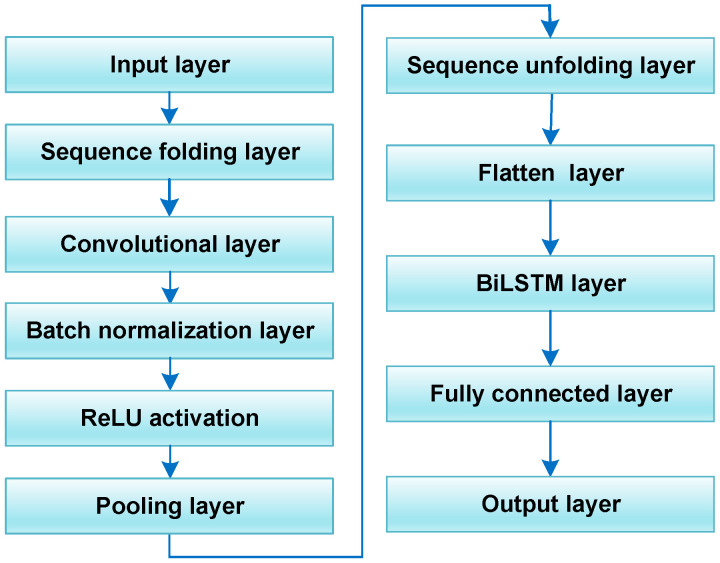
The structure of CNN–BiLSTM neural network.

Figure 4 illustrates the concrete structure of the CNN–BiLSTM prediction model. The model structure includes two main parts. Firstly, the CNN applies its unique structure to complete the convolutional pooling operation of input data, achieving data information mining and dimension reduction. Then, special gating units of the BiLSTM network handle the processed data, leveraging a large amount of information to conduct self-iterative training. During this process, the network learns and establishes a bidirectional temporal fitting relationship from previous data. The predicted values of the CNN–BiLSTM model are ultimately output by the output layer. This entire process encompasses the establishment of a predictive model for PV data.

### 2.2. Snake Optimization Algorithm

The snake optimization (SO) algorithm is a novel heuristic algorithm. This algorithm emulates the process of foraging, mating, and fighting of male and female snakes under conditions of food availability and temperature variations. Taking into account the snakes’ behavioral patterns, it is classified into two stages: the exploration phase and the exploitation phase [32].

#### 2.2.1. Initializing the Population

Similar to other heuristic algorithms, the optimization process of SO commences by creating a population that is uniformly distributed randomly. The initial population is calculated as follows:

(12) $$X_{i}=X_{min}+r\times (X_{max}-X_{min})$$

where $X_{i}$ indicates the location of the *i*-th individual, $X_{min}$ and $X_{max}$ indicate the lower and upper bounds of the population, respectively, and r is a random number between 0 and 1.

#### 2.2.2. Divide the Snakes into Equal Female and Male Groups

The SO algorithm splits the population equally into two main groups, male and female cohorts, as depicted by the following equation:

(13) $$N_{m}=N/2$$

(14) $$N_{f}=N-N_{m}$$

where *N* indicates the collective count of individuals within the population and $N_{m}$ and $N_{f}$ indicate the number of males and females in the population, respectively.

#### 2.2.3. Assess Each Group and Determine the Temperature and Amount of Food

Pick out the optimal individuals within every group and obtain the best male ($f_{best,m}$) and best female ($f_{best,f}$) as well as the location of food ($f_{food}$). Temperature (Temp) and food quantity (Q) are calculated as follows:

(15) $$Temp=exp(\frac{-t}{T})$$

(16) $$Q=c_{1}*exp(\frac{t-T}{T})$$

where *t* symbolizes the current iterations, while *T* indicates the maximum number of iterations, and $c_{1}$ = 0.5.

#### 2.2.4. Exploration Phase (No Food)

If Q < Threshold (0.25), the formula for updating the location of individual male and female snakes is as follows:

(17) $$X_{i,m}\left(t+1\right)=X_{rand,m}(t)\pm c_{2}\times A_{m}\times ((X_{max}-X_{min})\times rand+X_{min})$$

(18) $$X_{i,f}\left(t+1\right)=X_{rand,f}(t)\pm c_{2}\times A_{f}\times ((X_{max}-X_{min})\times rand+X_{min})$$

where $X_{i,m}$ and $X_{i,f}$ represent the locations of the ith male and female snakes, while $X_{rand,m}$ and $X_{rand,f}$ denote the positions of any randomly selected individual from the male and female snake populations, respectively, rand is a random number between 0 to 1, and $c_{2}$ = 0.05. The symbol “±” indicates the positive or negative sign, which is randomly determined in the calculation. $A_{m}$ and $A_{f}$ represent the hunting abilities of males and females for food, as shown in the following equation:

(19) $$A_{m}=exp(\frac{-f_{rand,m}}{f_{i,m}})$$

(20) $$A_{f}=exp(\frac{-f_{rand,f}}{f_{i,f}})$$

where $f_{rand,m}$ and $f_{rand,f}$, respectively, represent the fitness of $X_{rand,m}$ and $X_{rand,f}$, while $f_{i,m}$ and $f_{i,f}$ represent the fitness values of the *i*-th male snake and female snake.

#### 2.2.5. Exploitation Phase (Food Exists)

If Q > Threshold;

If the temperature > Threshold (0.6) (hot);

Snakes only move towards food:

(21) $$X_{i,j}\left(t+1\right)=X_{food}\pm c_{3}\times Temp\times rand\times (X_{food}-X_{i,j}\left(t\right))$$

where $X_{i,j}$ represents the location of either a male or female individual, while $X_{food}$ represents the optimal position for an individual, and $c_{3}$ = 2.

If the temperature < Threshold (0.6) (cold);

The snake will be in either a fight or mating mode.

Fight Mode:

(22) $$X_{i,m}\left(t+1\right)=X_{i,m}\left(t\right)+c_{3}\times FM\times rand\times (Q\times X_{best,f}-X_{i,m}\left(t\right))$$

(23) $$X_{i,f}\left(t+1\right)=X_{i,f}\left(t\right)+c_{3}\times MM\times rand\times (Q\times X_{best,m}-X_{i,f}\left(t\right))$$

where $X_{i,m}$ and $X_{i,f}$ represent the positions of the *i*th male and female individuals, respectively, while $X_{best,m}$ and $X_{best,f}$ denote the positions of the best individuals in the male and female populations. *FM* and *MM* refer to the combat abilities of male and female individuals, respectively, as shown by the following equation.

(24) $$FM=exp(\frac{-f_{best,f}}{f_{i}})$$

(25) $$MM=exp(\frac{-f_{best,m}}{f_{i}})$$

where $f_{best,m}$ and $f_{best,f}$ respectively, refer to the fitness values of the top individuals in the male and female populations, while $f_{i}$ represents the target fitness.

Mating Mode:

(26) $$X_{i,m}\left(t+1\right)=X_{i,m}\left(t\right)+c_{3}\times M_{m}\times rand\times (Q\times X_{i,f}(t)-X_{i,m}\left(t\right))$$

(27) $$X_{i,f}\left(t+1\right)=X_{i,f}\left(t\right)+c_{3}\times M_{f}\times rand\times (Q\times X_{i,m}(t)-X_{i,f}\left(t\right))$$

where $M_{m}$ and $M_{f}$ represent the mating competence of male and female individuals, respectively, as shown by the subsequent equation.

(28) $$M_{m}=exp(\frac{-f_{i,f}}{f_{i,m}})$$

(29) $$M_{f}=exp(\frac{-f_{i,m}}{f_{i,f}})$$

If the eggs hatch, they replace the lowest fitness male and female individuals.

(30) $$X_{worst,m}=X_{min}+rand\times (X_{max}-X_{min})$$

(31) $$X_{worst,f}=X_{min}+rand\times (X_{max}-X_{min})$$

where $X_{worst,m}$ and $X_{worst,f}$ indicate the location of the worst individual in the male group and female group, respectively.

### 2.3. Improved Snake Optimization Algorithm

This section will present the improvement methods of SO. This study improved the snake optimization algorithm in four aspects. Firstly, the initialization of snake populations utilizes the Tent chaotic mapping method to enhance randomness and diversity, thereby reducing uncertainty in the population initialization process. Secondly, by adjusting the food quantity threshold, the algorithm’s convergence speed can be improved by reducing the time spent in the exploration phase. Then, a lens imaging backward learning strategy is introduced to enable the algorithm to obtain dynamic and inverse solutions in lens backward learning, enhancing the global search ability. Finally, the optimal individual adaptive perturbation strategy is introduced to randomly perturb the position of the current optimal solution, preventing the algorithm from getting trapped in local optima.

#### 2.3.1. Tent Mapping Initialization

The quality of the population during the initialization phase directly determines the excellence of the algorithm, thus making it crucial for the algorithm [36,37]. The basic snake optimization algorithm usually employs a random initialization method to generate the initial population during the initialization phase. However, this method possesses a high degree of randomness and lacks diversity, resulting in the population being unable to evenly distribute within the search space. The Tent mapping is incorporated into the optimization procedure to elevate the performance of the snake optimization algorithm. The equation for the Tent mapping is presented below:

(32) $$z_{i+1}=\left\{\begin{array}{c} \frac{z_{i}}{\varepsilon },\;\;\;\;0\leq z_{i}\leq \varepsilon \\ \;\frac{1-z_{i}}{1-\varepsilon },\;\;\;\varepsilon <z_{i}\leq 1\;\; \end{array}\right.(i=1,2,\cdot \cdot \cdot )$$

where $z_{i}$ indicates the *i*-th chaotic value of the chaotic sequence, with $z_{i}$ ranging from 0 to 1. The control parameter “ε” ranges from 0 to 1, with a specific value of 0.6 selected in this article based on the simulation experiment results.

Based on Equation (32), the initial positions of individuals in the snake swarm based on the Tent chaotic map can be obtained as follows:

(33) $$X_{i}=X_{min}+z_{i}\left(X_{max}-X_{min}\right)(i=1,2,\cdot \cdot \cdot ,N_{p})$$

where $X_{min}$ is the lower limit of the solution and $X_{max}$ is the upper limit of the solution.

#### 2.3.2. Improvement of Food Quantity Threshold

**Figure 5:**
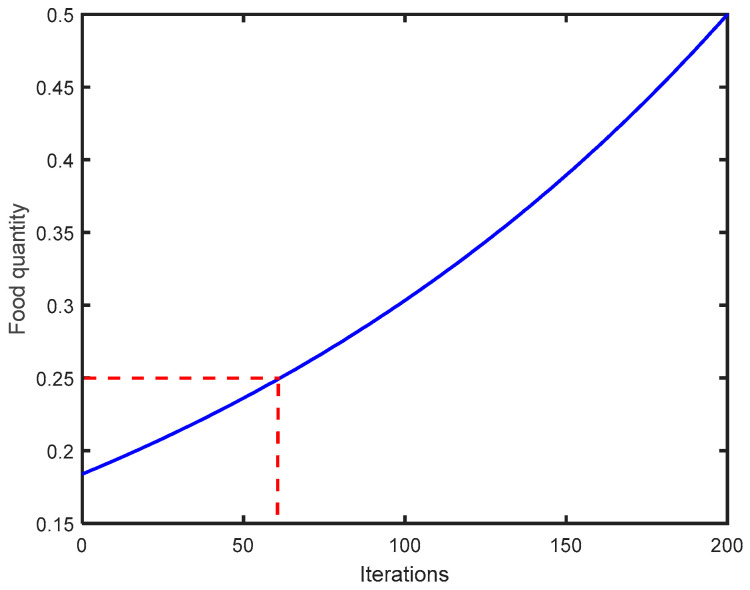
The curve of food quantity fluctuates with each iteration.

The convergence rate of SO is greatly affected by the food threshold. Figure 5 illustrates the correlation between the amount of food and the total number of iterations, assuming the maximum number for iterations is set at 200 in Equation (16).

It can be observed from Figure 5 that the amount of food is positively correlated with the number of iterations. Reducing the threshold Q for food can diminish the number of iterations required for global optimization search, thereby accelerating the convergence rate of the optimization process. To enhance the precision of PV power generation prediction without significantly affecting the algorithm’s global exploration capability, the food quantity threshold in the snake optimization algorithm has been adjusted from 0.25 to 0.2 through multiple experiments and adjustments.

#### 2.3.3. Lens Imaging Backward Learning Strategy

**Figure 6:**
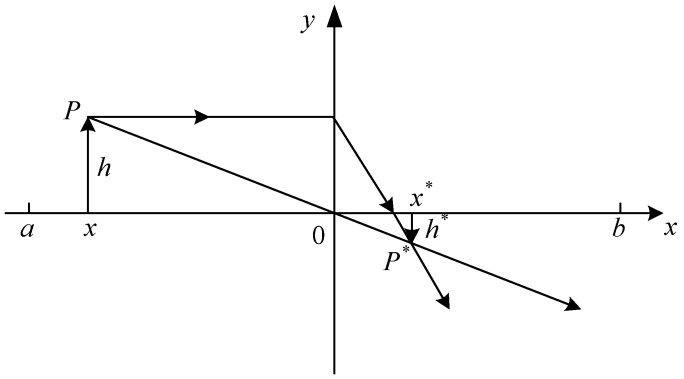
The schematic diagram of the lens imaging backward learning strategy.

Employing the strategy of backward learning in swarm intelligence, optimization algorithms can enhance the algorithm’s ability to achieve optimal solutions to a certain extent [38,39]. However, the backward solution obtained through backward learning is fixed. If an individual is already trapped in a local optimum and its backward solution is inferior to the current solution, the backward learning strategy cannot help the individual escape the local optimum. On the other hand, lens imaging backward learning can effectively address the aforementioned issue. The backward learning strategy for lens imaging is depicted in Figure 6.

Taking the one-dimensional space as an example, the search range for the solution is represented by [a, b], with the y-axis denoting the convex lens. This assumes the presence of an object P with a height of *h*, and its projection on the x-axis is denoted as *x*. When this object passes through a convex lens, it forms an inverted real image $P^{*}$ with a height of $h^{*}$ on the opposite side of the convex lens, and its projection on the x-axis is denoted as $x^{*}$. From the principles of convex lens imaging, it can be derived that:

(34) $$\frac{(a+b)/2-x}{x^{*}-(a+b)/2}=\frac{h}{h^{*}}$$

When *k* is equal to $h/h^{*}$, Equation (34) can be rewritten as:

(35) $$x^{*}=\frac{a+b}{2}+\frac{a+b}{2k}-\frac{x}{k}$$

Equation (35) is the inverse solution formula for the convex lens backward learning strategy. Equation (35) can be simplified as follows when *k* = 1:

(36) $$x^{*}=a+b-x$$

This equation represents the solving formula for backward learning.

From the aforementioned, it is evident that backward learning is a peculiar lens imaging backward learning, where a fixed backward solution is attained through backward learning. By adjusting the magnitude of *k*, dynamic variation of backward solutions can be achieved in lens backward learning, thereby further enhancing the algorithm’s optimization capability. The equation employed for calculating the value of *k* in this article is as follows:

(37) $$k={\left(1+{\left(\frac{t}{T}\right)}^{0.5}\right)}^{10}$$

#### 2.3.4. The Most Optimal Individual Adaptive Perturbation Strategy

This article introduces a variable mutation factor based on the number of iterations *t* as a system parameter to perform adaptive mutation on the optimal individual. The adaptive *t* distribution combines the advantages of the Gaussian distribution and the Cauchy distribution. When used as a mutation factor for adaptive perturbation on the optimal individual, it enhances the algorithm’s search capability and reduces the probability of getting trapped in local optima [40]. The specific equation is as follows:

(38) $${Best}_{i}^{’}={Best}_{i}+{Best}_{i}\cdot trnd(t)$$

where trnd(t) represents the *t*-distribution and ${Best}_{i}^{’}$ represents the mutated optimal individual position. When implementing adaptive perturbation, it is difficult to directly determine if the mutated individual is superior to the original individual. Hence, a greedy strategy is used to compare their fitness and select the optimal individual. The specific equation is:

(39) $${Best}_{new}=\left\{\begin{array}{c} {Best}_{i}^{’}\;\;\;if\;f({Best}_{i}^{’})\leq f({Best}_{i})\\ {Best}_{i}\;\;\;otherwise\;\;\;\;\;\;\;\;\;\;\;\; \end{array}\right.$$

where ${Best}_{new}$ refers to the optimized position of the selected individuals, and f(·) refers to the value of their fitness.

### 2.4. Multi-Strategy Improved Snake Optimization Algorithm Run Procedure

**Figure 7:**
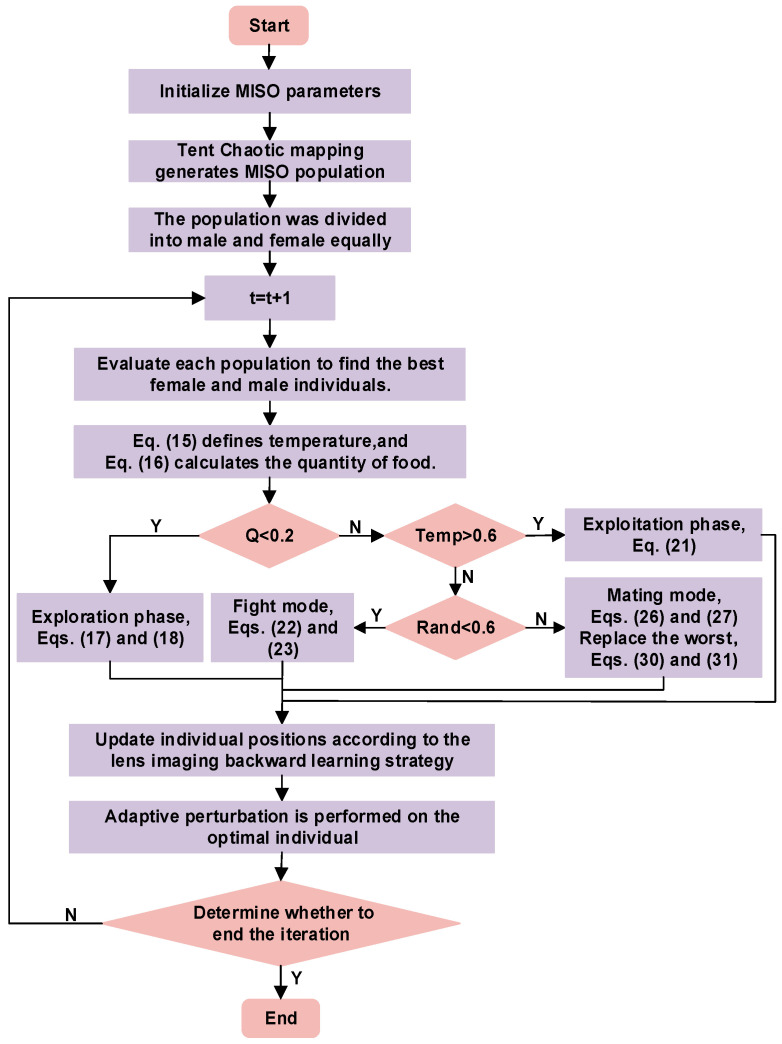
MISO flow chart.

The running procedure of the MISO algorithm unfolds in the subsequent manner, and the optimization flowchart is presented in Figure 7.

(1) Set the number of populations and the number of iterations.
(2) Initiate the population by generating initial solutions using the Tent chaotic mapping method.
(3) The population is classified into two categories, male and female, according to Equations (13) and (14). A fitness function is established, and the corresponding fitness values are calculated to identify the present optimal male and female individuals.
(4) The ambient temperature, denoted as Temp, and the quantity of food, denoted as Q, are defined according to Equations (15) and (16).
(5) It is determined whether the snake is foraging or engaged in fighting and mating based on the amount of food *Q* available. If food is scarce, the snake will search for it and update its individual position according to Equations (17) and (18).
(6) If food is plentiful and Temp > 0.6, the snake will only seek out food and consume existing food, updating its position according to (21).
(7) The snake individuals switch between combat mode and mating mode based on a random number *Rand*. During fight mode, their positions are updated using Equations (22) and (23), while during mating mode, their positions are updated using Equations (26) and (27). After the snake individuals engage in mating and their eggs hatch, the worst individuals are selected and replaced.
(8) Using a backward learning strategy based on lens imaging, the individual’s position is updated, and a new fitness value based on the updated position is calculated. Furthermore, the fitness values of the current male and female populations, as well as the global optimum, undergo updates.
(9) According to Equations (38) and (39), perform self-adaptive perturbation on the optimal individual.
(10) Determine whether the maximum number of iterations has been achieved. If so, terminate the iterative process and output the fitness value and position of the optimal individual. If not, proceed to the next iteration.

### 2.5. Establishment of Multi-Strategy Improved Snake Optimization Algorithm–Convolutional Neural Network–Bidirectional Long Short-Term Memory Network Prediction Model

**Figure 8:**
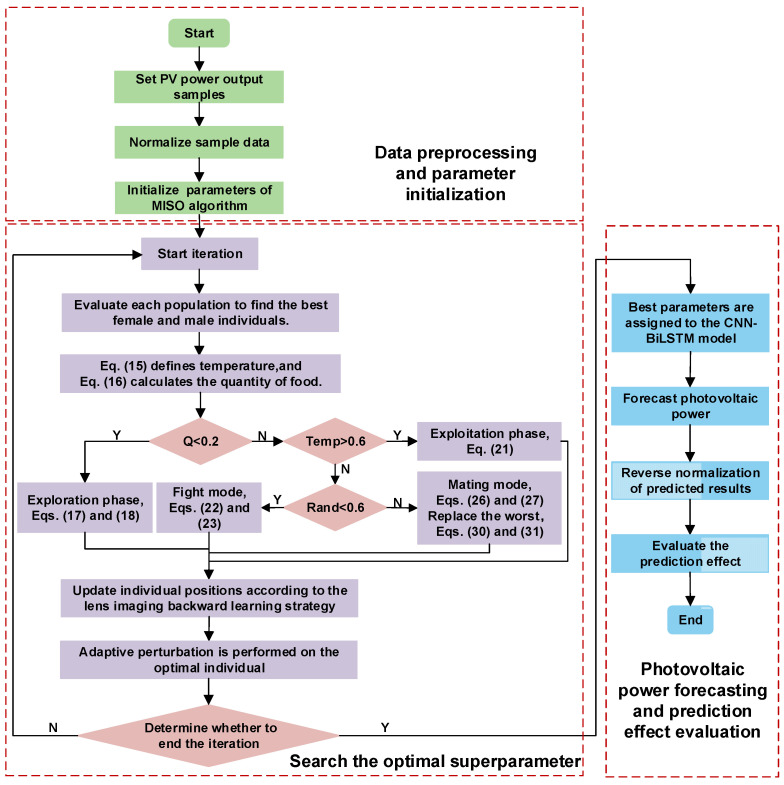
The optimization process of MISO–CNN–BiLSTM.

MISO can optimize the main parameters of CNN–BiLSTM, including learning rate, regularization coefficient, and number of hidden layer neurons, with good robustness and easy convergence. This article proposes a new PV power generation prediction model, MISO–CNN–BiLSTM. Figure 8 depicts the predictive procedure of the MISO–CNN–BiLSTM model and is displayed below:

(1) Determine the sample of PV output power.
(2) Normalize the sample data.
(3) Initialize the parameters of the MISO algorithm.
(4) The location update strategy of the MISO algorithm is utilized to update the locations of individual snakes.
(5) The hyperparameters of the CNN–BiLSTM model are optimized by the MISO algorithm.
(6) The trained MISO–CNN–BiLSTM model is employed to forecast PV power.
(7) Evaluate the predictive effect.

## 3. Analysis of Influencing Factors of Photovoltaic Output Power

### 3.1. Study of Power Output Curves of Photovoltaic Power under Different Weather Scenarios

**Figure 9:**
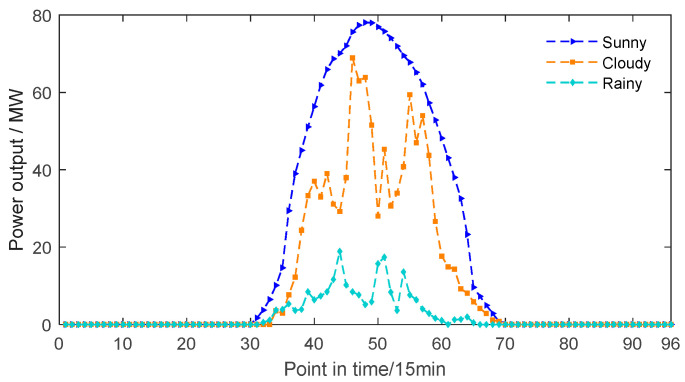
PV output power curve of three weather types.

Weather conditions possess a significant influence over PV power generation. To conduct a more comprehensive investigation into the influence of diverse weather scenarios on PV output power, actual output power data under three weather patterns—sunny, cloudy, and rainy—were chosen and analyzed from the collected sample data of PV power stations. The power output variations for the three weather conditions are depicted in Figure 9.

From Figure 9, it can be noticed that the PV output curves vary significantly among different weather patterns. During sunny days, the output power varies relatively smoothly throughout the day with few fluctuations, thus achieving the most optimal PV output. However, during cloudy weather, the unstable illumination leads to large fluctuations in output power throughout the day, resulting in an overall lower output power compared to sunny days. During rainy weather, the PV output efficiency reaches its minimum as the output power fluctuates significantly throughout the day, leading to an inadequate power generation effect.

### 3.2. K-Means Weather Clustering

**Figure 10:**
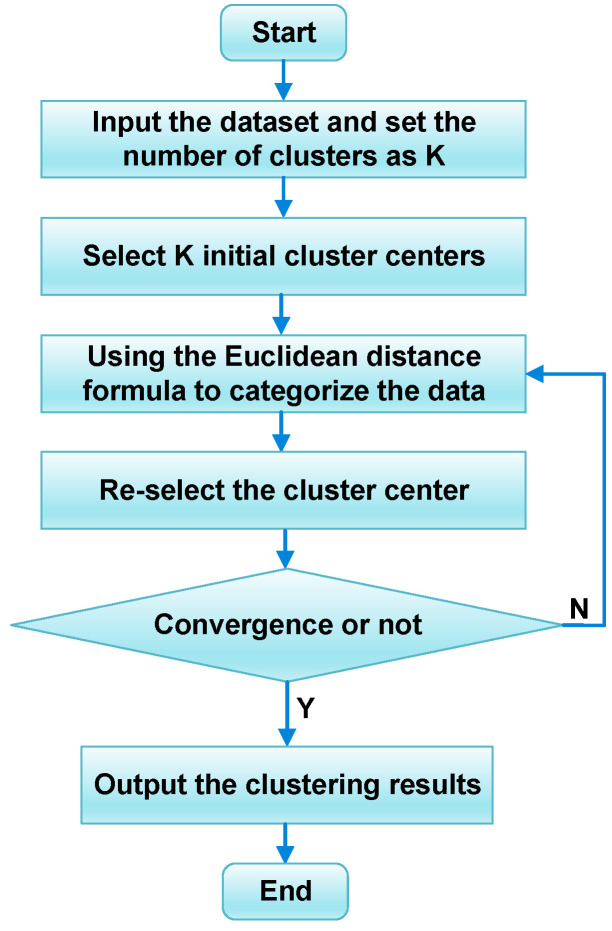
The flowchart of the K-means clustering algorithm.

In short-term PV power forecasting, the effectiveness of neural network prediction models can be greatly affected by significant differences between trained and predicted data, resulting in inaccurate predictions. Therefore, this study introduces the K-means clustering algorithm to categorize the weather and improve the forecast accuracy. The flowchart of the K-means clustering algorithm is depicted in Figure 10.

In the k-means clustering process, the data are first imported into the clustering model and divided into K categories according to the requirements of the dataset. The initial center points are then determined as K data points. Subsequently, the distances between the remaining data and the initial centers are computed and match every data point to the closest category. After computing the new clustering center points, the procedure is repeated until the objective function converges. The distance measure used for K-means clustering is the commonly used Euclidean distance, which is expressed as Equation (40).

(40) $$d=\sqrt{{\left(x_{i}-x_{i-1}\right)}^{2}-{\left(y_{i}-y_{i-1}\right)}^{2}}$$

where $x_{i}$ and $x_{i-1}$ represent the abscissa values of two randomly chosen points, while $y_{i}$ and $y_{i-1}$ denote the ordinate values of the same two points, and d denotes the Euclidean distance between these two points.

In this study, the average daily solar irradiance is set as the primary data for the clustering algorithm, with a value of K equal to 3. After iterative processing, we obtained three different weather categories, recorded as sunny, cloudy, and rainy based on the magnitude of irradiance. The range of average daily solar irradiance for sunny days is [222.968, 345.927] $W/m^{2}$, for cloudy days is [105.512, 216.937] $W/m^{2}$, and for rainy days is [5.452, 102.049] $W/m^{2}$.

### 3.3. The Influence of Different Meteorological Elements on Photovoltaic Power Output

**Figure 11:**
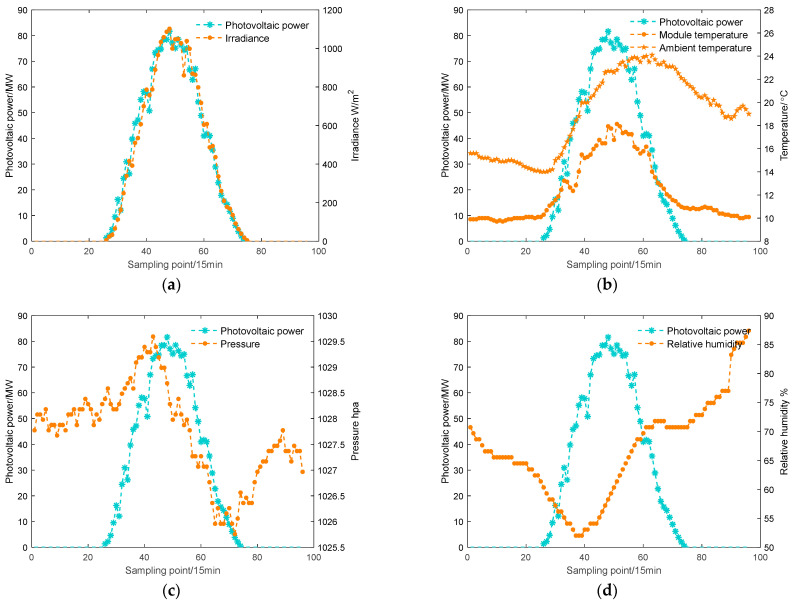
Relationship curves. (**a**) Irradiance and PV output power change curve; (**b**) temperature and PV output power change curve; (**c**) pressure and PV output power change curve; (**d**) relative humidity and PV output power change curve.

PV power is subject to numerous factors, which can be mainly classified into the internal parameters of the equipment in the PV power generation system and the external meteorological factors. Because the internal parameters of the PV system components are determined by the manufacturer, these parameters remain relatively stable once the PV power plant is installed. Hence, the solar power output is predominantly influenced by external environmental factors [41]. Based on historical data from 24 April 2020, the relationship curve between output power and irradiance, relative humidity, temperature, and pressure is plotted, as shown in Figure 11. The irradiance refers to the total solar radiation, including both direct and diffuse radiation. Direct irradiation is the radiant energy from the sun that directly reaches the ground, while diffused irradiation is the radiant energy from the sun that reaches the ground after being scattered by particles, molecules, etc. in the atmosphere. Under the obstructive effect of the atmosphere, the total radiation received by the ground will vary due to the influence of direct and diffused irradiation. Therefore, the irradiance studied in this paper refers to all the radiant energy from the sun.

From Figure 11, it is apparent that there is a strong and positive relationship between irradiance and the corresponding power output, where the strength of PV power increases as the irradiance rises and decreases with the reduction of irradiance. There exists a clear correlation between temperature and power, with the overall variation curve of PV output power showing consistency with temperature. Relative humidity and pressure, on the other hand, exhibit almost no correlation with power.

The aforementioned analysis has explored the diverse levels of correlation amid PV power output and several meteorological variables, including radiation intensity and pressure. However, these relationships are purely descriptive in nature. This study uses the Pearson correlation coefficient method to perform a quantitative analysis of the effect of meteorological factors on PV power, with the equation presented as follows:

(41) $$\rho_{x,y}=\frac{n\sum xy-\sum x\sum y}{\sqrt{n\sum x^{2}-{(\sum x)}^{2}}\sqrt{n\sum y^{2}-{\left(y\right)}^{2}}}$$

where *x* and *y* are correlated variables, with *n* being the total sample size. *x* represents weather factors and *y* represents the output power of PV cells. $\rho_{x,y}$ denotes the correlation coefficient.

Table 1 illustrates the implications of Pearson’s coefficient [42]. When $\rho_{x,y}$ is greater than 0, it denotes a positive correlation. When $\rho_{x,y}$ is equal to 0, it signifies no linear correlation. Conversely, when $\rho_{x,y}$ is less than 0, it indicates a negative correlation.

Pearson correlation analysis was conducted using the data from the entire month of April, 2020, and the results are listed in Table 2.

From Table 2, it is apparent that the correlation coefficient between PV power and radiation intensity reaches 0.978, indicating a strong positive relationship. Additionally, there exists a moderate positive correlation with temperature, encompassing both environmental and component temperatures. Conversely, the correlation between pressure and relative humidity is relatively weak. Therefore, this study selects irradiance, ambient temperature, and component temperature as the inputs for the model, with PV output power as the output.

## 4. Simulation Experiment Analysis

### 4.1. Optimizer Performance Analysis

To verify the correctness of the strategy selection for the MISO algorithm optimization, six classic benchmark test functions were selected to assess the optimization performance of MISO, as listed in Table 3. Among the six test functions, $f_{1}\left(x\right)-f_{3}(x)$ are unimodal test functions employed to examine the algorithm’s convergence ability and solution accuracy; $f_{4}\left(x\right)-f_{6}(x)$ are multimodal test functions, which can effectively test the algorithm’s global exploration capability. By utilizing these different types of test functions, the optimization performance of the MISO algorithm can be thoroughly validated.

To comprehensively validate the efficacy of the MISO algorithm put forth in this study, we selected the GWO algorithm, WOA, and SO algorithm for comparison. These algorithms have been proven to possess excellent optimization capabilities. To accurately assess the performance of the MISO algorithm versus the contrastive algorithms, a unified population size of 30, a function dimension of 30, and a maximum of 500 iterations were set for all algorithms. Each algorithm was independently executed 30 times. Table 4 shows the parameter settings of the comparison algorithm, and Table 5 shows the experimental results.

Table 5 indicates that MISO exhibits remarkable performance advantages for unimodal test functions. When solving functions $f_{1}\left(x\right)$, $f_{2}\left(x\right)$, and $f_{3}\left(x\right)$, the MISO algorithm achieves the theoretical optimum, which is far superior to SO and other compared algorithms. Furthermore, compared with the three algorithms, the MISO algorithm has the smallest standard deviation, indicating that MISO algorithm has the best exploration ability and stability.

Regarding the multimodal test functions, the MISO algorithm achieved the theoretically optimal value when solving for function $f_{4}\left(x\right)$. Meanwhile, for functions $f_{5}\left(x\right)$ and $f_{6}\left(x\right)$, none of the algorithms reached the theoretical optimal value. However, the MISO algorithm still had the highest search precision compared to other algorithms. These results indicate that the MISO algorithm possesses both strong global exploration and local optima avoidance abilities, as well as high optimization stability.

### 4.2. Predictive Result Analysis

The data for this study were from the Taiyangshan PV Power Station in Ningxia, China, in 2020, and samples were taken every 15 min. To assess the predictive precision of the established model, this study used k-means clustering to divide weather scenarios into three small sample datasets: sunny, cloudy, and rainy, based on the size of irradiance. Then, from January to June, 30 days of data were selected for simulation analysis for each weather type, and 2784 samples were allocated as the training set and 96 samples as the testing set.

The MISO–CNN–BiLSTM model was utilized to predict PV power. Additionally, the comparison models employed were BP, LSTM, BiLSTM, CNN–BiLSTM, and SO–CNN–BiLSTM. Furthermore, within this study, the error evaluation metrics selected were mean absolute error (*MAE*), root mean squared error (*RMSE*), and coefficient of determination ($R^{2}$). The computation expressions are as follows:

(42) $$MAE=\frac{1}{n}\sum_{i=1}^{n}\left|y_{i}-y_{i}^{*}\right|$$

(43) $$RMSE=\sqrt{\frac{1}{n}\sum_{i=1}^{n}{\left(y_{i}-y_{i}^{*}\right)}^{2}}$$

(44) $$R^{2}=1-\frac{\sum_{i=1}^{n}{\left(y_{i}-y_{i}^{*}\right)}^{2}}{\sum_{i=1}^{n}(y_{i}-\bar{y_{i}})}$$

where *n* refers to the number of test sets, $y_{i}$ refers to the actual PV power value, $y_{i}^{*}$ refers to the predicted value of the model, and $\bar{y_{i}}$ denotes the average value of the PV power data set.

#### 4.2.1. Prediction Results in Sunny Weather

**Figure 12:**
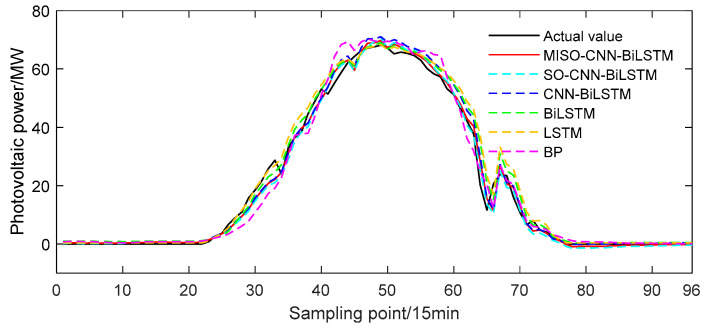
Predicted power output curves on a sunny day.

The MISO–CNN–BiLSTM model was validated using solar power output data on a sunny day, specifically on 13 June 2020. The training set consisted of sunny day power output data from the previous 29 days leading up to June 13, while the solar power output on June 13 itself served as the test set. The predicted outcomes of the MISO–CNN–BiLSTM and the comparison models can be seen in Figure 12.

From Figure 12, it can be observed that during sunny weather, the general trend of the PV output power curve was stable, exhibiting remarkable regularity. This was due to the steady variation of various meteorological factors, resulting in a slow change in PV output power with variance in solar irradiance and temperature under sunny circumstances. The changing trends of the five predicted curves were generally consistent with the actual values. Among them, the MISO–CNN–BiLSTM model provided the closest prediction results to the actual values, indicating its superior predictive performance. Compared to the other models, the BP model’s output power curve deviated the most from the actual values, highlighting its poor predictive capability.

**Figure 13:**
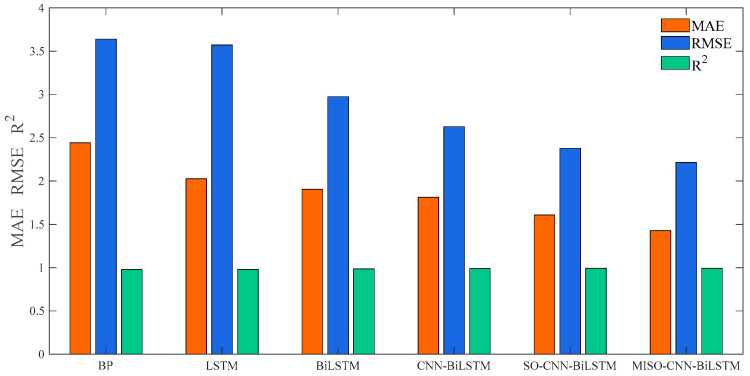
Comparison of models for sunny weather.

In order to observe the prediction outcomes more directly, MAE, RMSE, and $R^{2}$ were utilized to assess the predictive precision of the six models. The evaluation findings are listed in Table 6 and Figure 13. The MAE for the MISO–CNN–BiLSTM method is 1.4269, the RMSE is 2.213, and $R^{2}$ is 0.99216. All evaluation metrics outperformed those of the other comparative models. In general, the MISO–CNN–BiLSTM model produced the most optimal prediction results, thus confirming the efficacy of the established prediction model.

#### 4.2.2. Prediction Results in Cloudy Weather

**Figure 14:**
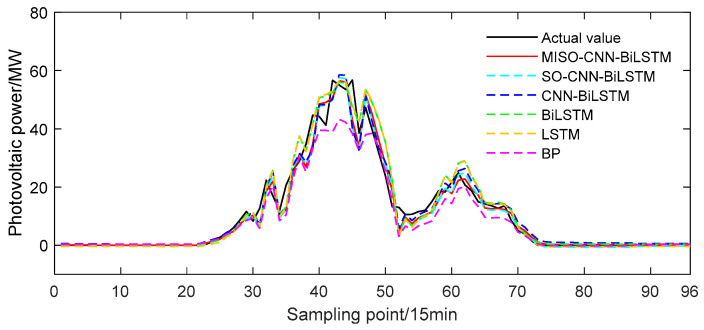
Predicted power output curves on cloudy day.

The MISO–CNN–BiLSTM model was evaluated using cloudy power output data on 23 June 2020. The training set consisted of cloudy power output data from the previous 29 days leading up to 23 June 2020, while the PV power output on 23 June 2020 was used as the test set. The predictive outcomes of the MISO–CNN–BiLSTM and the comparison models are depicted in Figure 14.

From Figure 14, it is evident that during cloudy conditions, there was significant volatility in the PV output curve. Moreover, there were noticeable variations in the predictions of different forecasting models during certain time periods, indicating distinct discrepancies. In terms of overall prediction accuracy, the MISO–CNN–BiLSTM model outperformed other models, as its curve closely aligned with the actual values.

**Figure 15:**
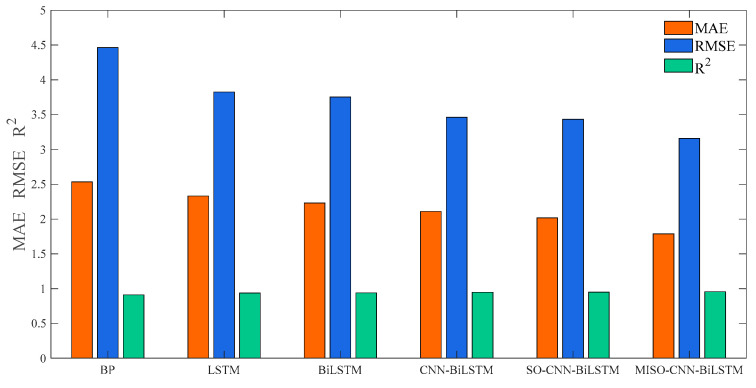
Comparison of models for cloudy weather.

Table 7 and Figure 15 present the evaluation metrics for six weather forecasting models under cloudy conditions. The MAE for the MISO–CNN–BiLSTM model is 1.7877, the RMSE is 3.1595, and the $R^{2}$ is 0.95772. All these evaluation metrics outperformed other comparative models, thereby substantiating the efficacy of the established forecasting model.

#### 4.2.3. Prediction Results in Rainy Weather

**Figure 16:**
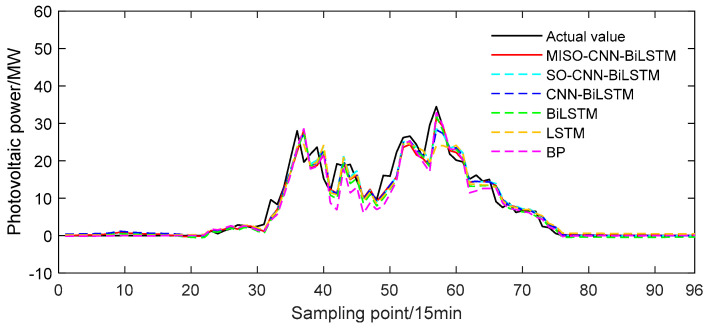
Predicted power output curves on rainy day.

The MISO–CNN–BiLSTM model was evaluated using power output data on a rainy day, specifically on 24 June 2020. The training set consisted of power output data from the preceding 29 days, while the test set included the PV power output on 24 June 2020. The predictive outcomes of the MISO–CNN–BiLSTM and the comparison models are displayed in Figure 16.

From Figure 16, it is apparent that during rainy weather, the PV power curve fluctuated greatly and had weaker regularity, leading to less satisfactory prediction results of the model and lower accuracy compared to sunny and cloudy days. However, the MISO–CNN–BiLSTM model showed the closest proximity between its forecasts and the factual measurements for all the models, which demonstrated the validity of the established model under cloudy and rainy conditions.

**Figure 17:**
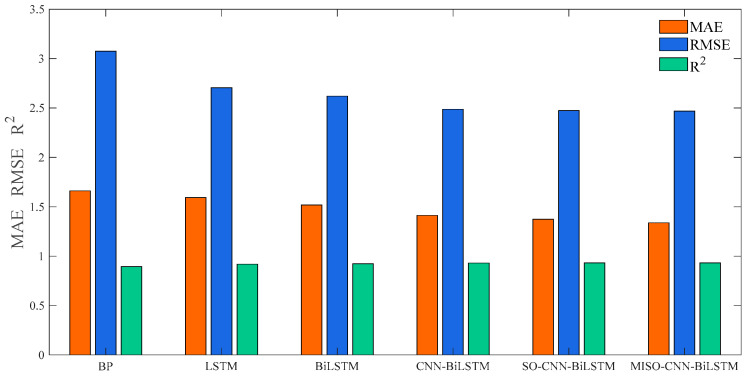
Comparison of models for rainy weather.

Table 8 and Figure 17 present the evaluation metrics of six weather models for rainy days. The MAE for the MISO–CNN–BiLSTM model is 1.3374, the RMSE is 2.4689, and the $R^{2}$ is 0.93163. The assessment indicators of the MISO–CNN–BiLSTM model surpassed those of the other comparative models, thereby validating the efficacy of the established predictive model in this paper.

## 5. Conclusions

Due to the inherent uncertainty in PV power forecasting, particularly in situations with unpredictable weather changes, the precision of electricity predictions has become a significant technical challenge. This article employs K-means clustering to classify historical PV data, resulting in three distinct subsets: sunny, cloudy, and rainy. Based on these subsets, the corresponding PV power generation for distinct weather scenarios is forecasted. To enhance the precision of PV power prediction under varying weather types, this study utilizes the MISO–CNN–BiLSTM model. The empirical findings evince that the MISO–CNN–BiLSTM model surpasses the SO–CNN–BiLSTM, CNN–BiLSTM, BiLSTM, LSTM, and BP models in predicting performance. The conclusions of this research are as follows:

(1) Combining multiple enhancement techniques enhances the optimization performance of SO. The integration of the original SO with the Tent chaotic initialization, lens imaging reverse learning strategy, and optimal individual adaptive perturbation strategy significantly improves the overall performance of MISO.
(2) The simulation findings demonstrate that the established model has excellent predictive prowess. In various weather conditions, the MISO–CNN–BiLSTM model demonstrates significantly lower MAE and RMSE values in comparison to the other models presented in this research, providing evidence of its high prediction accuracy. Furthermore, the $R^{2}$ values of the MISO–CNN–BiLSTM model surpass those of other models mentioned in this paper, substantiating its superiority and reliability.
(3) The MISO–CNN–BiLSTM model can accurately forecast PV power, which is helpful for power grid system planning and dispatching and reduces the dispatching cost of the power system.

The MISO–CNN–BiLSTM PV power prediction model proposed by this research can achieve accurate prediction of PV output power under different weather scenarios. This contributes to enhancing the utilization efficiency of renewable energy generation, ensuring the security of renewable energy power systems. Moreover, it plays a decisive role in advancing the growth of the renewable energy sector. In addition to PV prediction, the model can also be used for power prediction of other similar renewable energy sources and may become a universal renewable energy power prediction method, which can promote the wider use of renewable energy.

This study has limitations. Although this study provides forecasts for short-term PV generation across three distinct weather conditions, it overlooks the consideration of numerous extreme weather phenomena such as rainstorms, snowstorms, sandstorms, haze, etc. In the future, research should be conducted on the power prediction of PV generation under inclement meteorological conditions, so as to enhance the dependability of the prediction model.

## Figures and Tables

**Table 1:** Correlation coefficient corresponds to the degree of correlation.

$\left|\rho_{x,y}\right|$	Degree of Correlation
0.0–0.2	Exceedingly less or no correlation
0.2–0.4	Weak correlation
0.4–0.6	Moderate correlation
0.6–0.8	Strong correlation
0.8–1.0	Extremely strong correlation

**Table 2:** Pearson correlation analysis results.

Attributes	$\rho_{x,y}$
Radiation intensity	0.978
Component temperature	0.484
Ambient temperature	0.518
Pressure	−0.094
Relative humidity	0.075

**Table 3:** Test functions.

Functions	Dimension	Range	Optimum
$f_{1}(x)=\sum_{i=1}^{n}x_{i}^{2}$	30	[−100, 100]	0
$f_{2}(x)=\sum_{i=1}^{n}\left|x_{i}\right|+\prod_{i=1}^{n}\left|x_{i}\right|$	30	[−10, 10]	0
$f_{3}(x)=\sum_{i=1}^{n}(\sum_{j=1}^{i}x_{j}{)}^{2}$	30	[−100, 100]	0
$f_{4}(x)=\sum_{i=1}^{n}[x_{i}^{2}-10\cos(2\pi x_{i})+10]$	30	[−5.12, 5.12]	0
$f_{5}(x)=-20\exp(-0.2\sqrt{\frac{1}{n}\sum_{i=1}^{n}x_{i}^{2}})-\exp(\frac{1}{n}\sum_{i=1}^{n}\cos(2\pi x_{i}))+20+e$	30	[−32, 32]	0
$\begin{array}{l} f_{6}(x)=\frac{\pi }{n}\left\{10\right.\sin(\pi y_{1})+\sum_{i=1}^{n-1}{\left(y_{i}-1\right)}^{2}[1+10\sin^{2}(\pi y_{i+1})]\\ +(y_{n}-1\left.{)}^{2}\right\}+\sum_{i=1}^{n}u(x_{i},10,100,4)\\ y_{i}=1+\frac{x_{i}+1}{4},u(x_{i},a,k,m)=\left\{\begin{array}{l} k{\left(x_{i}-a\right)}^{m},x_{i}>a\\ 0,-a<x_{i}<a\\ k{\left(-x_{i}-a\right)}^{m},x_{i}<-a \end{array}\right. \end{array}$	30	[−50, 50]	0

**Table 4:** Setting parameters of GWO, WOA, SO, and MISO.

Algorithm	Parameters
GWO	a = 2~0
WOA	a = 2~0, b = 1
SO	c1 = 0.5, c2 = 0.05, c3 = 2
MISO	c1 = 0.5, c2 = 0.05, c3 = 2

**Table 5:** Test results.

Functions	Algorithms	Optimal	Worst Value	Average	Standard Deviation
$f_{1}\left(x\right)$	GWO	3.99 × 10^−29^	1.34 × 10^−27^	4.12 × 10^−28^	5.52 × 10^−28^
	WOA	2.10 × 10^−81^	1.45 × 10^−72^	2.91 × 10^−73^	6.50 × 10^−73^
	SO	6.07 × 10^−99^	1.14 × 10^−94^	2.54 × 10^−95^	4.95 × 10^−95^
	**MISO**	**0**	**0**	**0**	**0**
$f_{2}\left(x\right)$	GWO	3.55 × 10^−17^	1.27 × 10^−16^	6.71 × 10^−17^	4.06 × 10^−17^
	WOA	3.16 × 10^−55^	3.10 × 10^−49^	6.21 × 10^−50^	1.39 × 10^−49^
	SO	7.55 × 10^−45^	1.06 × 10^−42^	4.39 × 10^−43^	5.53 × 10^−43^
	**MISO**	**0**	**0**	**0**	**0**
$f_{3}\left(x\right)$	GWO	4.84 × 10^−7^	4.70 × 10^−4^	1.69 × 10^−4^	2.32 × 10^−4^
	WOA	2.70 × 10^4^	6.10 × 10^4^	4.14 × 10^4^	1.33 × 10^4^
	SO	1.26 × 10^−64^	2.61 × 10^−59^	5.85 × 10^−60^	1.14 × 10^−59^
	**MISO**	**0**	**0**	**0**	**0**
$f_{4}\left(x\right)$	GWO	5.68 × 10^−14^	13.85	3.80	6.04
	WOA	0	0	0	0
	SO	45.33	78.78	66.25	13.24
	**MISO**	**0**	**0**	**0**	**0**
$f_{5}\left(x\right)$	GWO	6.84 × 10^−14^	1.25 × 10^−13^	9.82 × 10^−14^	2.36 × 10^−14^
	WOA	8.88 × 10^−16^	7.99 × 10^−15^	5.86 × 10^−1^	3.18 × 10^−15^
	SO	4.44 × 10^−15^	2.90	0.58	1.30
	**MISO**	**8.88 × 10^−16^**	**8.88 × 10^−16^**	**8.88 × 10^−16^**	**0**
$f_{6}\left(x\right)$	GWO	0.03	7.91 × 10^−2^	4.84 × 10^−2^	2.07 × 10^−2^
	WOA	7.40 × 10^−3^	4.93 × 10^−2^	2.12 × 10^−2^	1.76 × 10^−2^
	SO	8.34 × 10^−1^	8.68	4.20	3.61
	**MISO**	**1.53 × 10^−5^**	**2.75 × 10^−4^**	**1.15 × 10^−5^**	**1.00 × 10^−4^**

**Table 6:** Predicted results table under sunny weather conditions.

Models	MAE	RMSE	R^2^
BP	2.4406	3.6391	0.97881
LSTM	2.0254	3.5709	0.9796
BiLSTM	1.9034	2.9728	0.98586
CNN–BiLSTM	1.813	2.6278	0.98895
SO–CNN–BiLSTM	1.6091	2.3773	0.99096
MISO–CNN–BiLSTM	1.4269	2.213	0.99216

**Table 7:** Predicted results table under cloudy weather conditions.

Models	MAE	RMSE	R^2^
BP	2.534	4.4629	0.91139
LSTM	2.3306	3.8253	0.93802
BiLSTM	2.2299	3.7537	0.94032
CNN–BiLSTM	2.1101	3.4632	0.9492
SO–CNN–BiLSTM	2.0159	3.4332	0.95007
MISO–CNN–BiLSTM	1.7877	3.1595	0.95772

**Table 8:** Predicted results table under rainy weather conditions.

Models	MAE	RMSE	R^2^
BP	1.6608	3.0752	0.89392
LSTM	1.5929	2.7063	0.91785
BiLSTM	1.5173	2.6198	0.92301
CNN–BiLSTM	1.4119	2.4873	0.9306
SO–CNN–BiLSTM	1.3728	2.4742	0.93133
MISO–CNN–BiLSTM	1.3374	2.4689	0.93163

## Data Availability

The data presented in this study are available on request from the corresponding author. The data are not publicly available due to privacy.

## Abbreviations

PV	photovoltaic
SVM	support vector machine
ELM	extreme learning machine
LSTM	long short-term memory
DA	dragonfly algorithm
PSO	particle swarm optimization
MBES	improved condor search algorithm
CNN	convolutional neural network
BiLSTM	bidirectional long short-term memory
SO	snake optimization
MISO	multi-strategy improved snake optimization
GWO	grey wolf optimizer
WOA	whale optimization algorithm
RNN	recurrent neural network
BP	back propagation
MAE	mean absolute error
RMSE	root mean squared error
R ^2^	coefficient of determination
